# Hospital Volume and Post‐Hepatectomy Liver Failure After Major Hepatectomy

**DOI:** 10.1002/jso.70280

**Published:** 2026-05-06

**Authors:** Xane D. Peters, Brian C. Brajcich, Bona Ko, Catherine Valukas, Lauren M. Janczewski, Clifford Y. Ko, Ryan P. Merkow, Henry A. Pitt, David J. Bentrem

**Affiliations:** ^1^ Department of Surgery, Northwestern Quality Improvement Research and Education in Surgery Chicago Illinois USA; ^2^ American College of Surgeons Division of Research and Optimal Patient Care Chicago Illinois USA; ^3^ Department of Surgery Loyola University Medical Center Maywood Illinois USA; ^4^ Department of Surgery Chicago Northwestern University Illinois USA; ^5^ Department of Surgery Stanford University Medicine Palo Alto California USA; ^6^ Department of Surgery University of California Los Angeles David Geffen School of Medicine, VA Greater Los Angeles Healthcare System Los Angeles California USA; ^7^ Department of Surgery University of Chicago Chicago Illinois USA; ^8^ Rutgers Cancer Institute of New Jersey New Brunswick New Jersey USA; ^9^ Jesse Brown Veterans Affairs Medical Center Chicago Illinois USA

**Keywords:** hepatectomy, hepatobiliary, hospital volume, liver failure, surgical quality

## Abstract

**Background:**

Post‐hepatectomy liver failure (PHLF) following major hepatectomy (MH) increases the risk of morbidity and death. The relationship between institutional MH volume, PHLF, and outcomes is not well characterized.

**Methods:**

Adults undergoing MH from 2014 to 2021 in the ACS NSQIP hepatectomy‐targeted database were included. Rates of PHLF were compared based on hospital‐level annual MH volume. Multivariable logistic regression evaluated the association between volume, PHLF grade, and outcomes.

**Results:**

Across 11,167 patients, PHLF incidence was 3.7% in low‐volume, 5.5% in low‐medium volume, 6.9% in medium‐high volume, 11.8% in high‐volume centers (*p* < 0.001). The adjusted odds ratio (aOR) for morbidity in grade B/C PHLF compared to those without PHLF was elevated in both lower‐volume centers (quartiles 1–3), (11.2 [7.04–17.70]) and in high‐volume centers, (8.47 [6.06–11.85]).

**Conclusion:**

Higher annual major hepatectomy volume is associated with increased PHLF, which may be a function of complex disease treated at these institutions. PHLF precedes other adverse events affecting both high and low volume institutions. PHLF is an important target for quality improvement.

## 1 Introduction

Liver resection is the mainstay of treatment for both primary and secondary liver tumors [1, 2, 3, 4], conducted mainly for colorectal liver metastasis (CRLM) in Western countries [1, 5]. The liver's unique ability to regenerate allows for large resections that place the patient at risk for postoperative insufficiency and failure before sufficient regeneration occurs [6, 7]. With modern technical developments and improved perioperative management, these procedures have become increasingly available [8], and mortality associated with liver resections has declined to 3%‐5% at high‐volume centers [1, 9, 10, 11, 12, 13, 14]. Nevertheless, post‐hepatectomy liver failure (PHLF) remains a major contributor to morbidity and is the leading cause of mortality after liver resections for a variety of indications, including hepatocellular carcinoma (HCC), biliary tumors, and CRLM [4, 15, 16, 17, 18].

The reported incidence of PHLF is variable within the literature, ranging from 1% to 34% [16, 19, 20, 21, 22, 23, 24, 25, 26]. A variety of factors clinical factors, contribute to clinically significant PHLF, including the extent of liver resection [27, 28]. Due to an absent universally accepted definition of PHLF, in 2011, the International Study Group of Liver Surgery (ISGLS) published a consensus‐based definition of PHLF alongside a grading scale aimed to predict postoperative prognosis [29]. The association of this definition with perioperative mortality has been reported in subsequent studies [30, 31].

The effect of surgical volume status on hospital quality metrics has been thoroughly discussed over the past several decades, supporting improved outcomes at high volume surgical centers [32, 33, 34], additionally reported in patients undergoing liver resection [1, 5]. However, the literature linking surgical volume with hepatectomy outcomes has largely been focused on mortality and length of stay (LOS) [1, 34, 35]. Our goals in this study were 1.) determine whether the incidence of PHLF differs based on hospital major hepatectomy (MH) volume, and 2.) evaluate the relationship between PHLF and other 30‐day outcomes at both high and low‐volume centers.

## 2 Materials and Methods

### 2.1 Data Source

This retrospective cohort study analyzed data from the American College of Surgeons National Surgical Quality Improvement Database (ACS NSQIP). The ACS NSQIP has been previously described [36, 37, 38]. Briefly, the ACS NSQIP is a multi‐institution clinical data registry that collects perioperative data regarding patient demographics, surgical details, and postoperative events to provide risk‐adjusted information on surgical outcomes and inform quality improvement initiatives. This data is abstracted from patient charts via professionally trained auditors. The ACS NSQIP offers a variety of ‘procedure targeted’ datasets that are tailored to specific procedure groupings. The procedure targeted hepatectomy dataset was made available by the ACS NSQIP in 2014, including 30 variables relevant to this procedure. Data from this ACS NSQIP hepatectomy dataset from 2014 through 2021 were used to identify patients for this study. This manuscript reports findings in accordance with STROBE guidelines for observational studies [39].

### 2.2 Inclusion and Exclusion

Adult patients who underwent elective MH were identified using the following Current Procedure Terminology (CPT) codes: 47,122 (Trisegmentectomy), 47,125 (Total Left Hepatectomy), and 47,130 (Total Right Hepatectomy). Patients undergoing non‐major hepatectomy ( < 3 segments) and those undergoing emergent hepatectomy were excluded (*n* = 78).

### 2.3 Volume Tier Assignment

Hospitals were defined solely by the volume of major hepatectomies performed annually. Operative volume has been previously demonstrated to be associated with additional specialized services, specifically for hepaticopancreaticobiliary surgery [40]. The ACS NSQIP dataset is a sampled clinical registry designed to balance representativeness with feasibility of data capture and data quality. Sampling within this clinical registry cycles over 8 days (Monday to Monday), with a fixed number of eligible major cases eligible for inclusion. Notably, small, low‐volume, and rural hospitals may capture 100% of all eligible major cases [41]. Quartiles were established using annual volume distributions from within this dataset. The average annual volume was calculated for each hospital by dividing total number of major hepatectomies from 2014 through 2021 by the number of non‐zero years of NSQIP hepatectomy data. Hospitals were placed in quartiles (Q1–Q4) based on their average annual MH volume. Hospitals in Q1–Q3 (“low‐volume”) were grouped for comparison with Q4 (“high‐volume”).

### 2.4 Predictor Variables

Patient factors included age, biologic sex, and race. Race category included White, Black or African American, and Asian/Other. The ‘Other’ race category included patients whose self‐identified race was unknown, other, or multiracial and was not independently evaluated due to sample size. Data reflecting disease state included American Society of Anesthesiologists (ASA) classification, presence of a pre‐existing bleeding disorder, ascites, preoperative bilirubin, aspartate aminotransferase (AST), and albumin. Hepatectomy‐specific variables included liver texture (normal, abnormal, unknown), tumor size, tumor pathology, biliary stent placement, biliary reconstruction, pre‐operative portal vein embolization, extent of liver resection (left hepatectomy, trisegmentectomy, right hepatectomy), and concurrent colorectal resection. Abnormal liver texture included those documented as fatty, congested, cirrhotic, or fibrosed. Tumor pathology included HCC, cholangiocarcinoma, other primary tumor, secondary metastasis, or unknown. Patients in which preoperative stent placement or intraoperative hepaticojejunostomy was unknown were grouped with the ‘no’ category due to insufficient numbers to be analyzed independently. Concurrent colorectal liver resections were identified by concurrent procedural terminology (CPT) codes (Supplement A).

### 2.5 Outcomes

The primary outcomes of this study were PHLF and morbidity after PHLF. Within the ACS NSQIP hepatectomy dataset, PHLF is defined based on ISGLS criteria: postoperatively acquired deterioration in the ability of the liver to maintain its synthetic, excretory, and detoxifying functions; characterized by an increase in INR and hyperbilirubinemia on or after postoperative day (POD) 5 and graded by severity (A, B, or C). Secondary outcomes included 30‐day mortality, 30‐day readmission, inpatient length of stay (LOS), return to the operating room (ROR), and need for invasive postoperative interventions (percutaneous drain placement, stent placement, or angiography/embolization). Within the ACS NSQIP, the composite 30‐day morbidity outcome is defined by the presence of Superficial Incisional Surgical Site Infection (SSI), Deep Incisional SSI, Organ/Space SSI, Wound Disruption, Pneumonia, Unplanned Intubation, on Ventilator > 48 h, Progressive Renal Insufficiency, Acute Renal Failure, Urinary Tract Infection, Stroke/Cerebral Vascular Accident, Cardiac Arrest, Myocardial Infarction, or Systemic Sepsis (Sepsis or Septic Shock). Given the comparison of separate models for high and low volume hospitals, non‐overlapping 95% confidence intervals were interpreted as statistically significant differences between models.

### 2.6 Post Hepatectomy Liver Failure

Post Hepatectomy Liver Failure was analyzed in two ways: as an outcome variable in logistic regression models comparing odds of PHLF at low volume versus high volume centers, and as a predictor variable for 30‐day mortality and subsequent adverse postoperative events.

### 2.7 Statistical Analyses

Unadjusted Chi squared analyses were used to compare predictor variables with the outcomes of interest. Associations between predictor variables and outcomes were further elucidated via multivariable logistic regression. Logistic regression models were constructed separately for each outcome of interest, and variables were chosen for inclusion in these models based on prior literature and clinical face validity [42, 43]. Observations were clustered at the hospital level to provide robust standard error estimates, accounting for non‐independence of observations within hospitals. All statistical analyses were conducted using SAS version 9.4 (SAS Institute).

## 3 Results

Across 176 participating hospitals, 11,167 MHs performed from 2014 through 2021 were eligible for inclusion. Most resections were right hepatectomies (46.7%), and preoperative stent placement (10.2%) and portal vein embolization (8.6%) were infrequent. Most tumors were secondary metastatic tumors (42.3%), and tumor size was unreported in 59.4% of cases. Very few patients (2.0%) underwent concurrent colorectal resection (Table 1).

**Table 1:** Cohort characteristics by grade of Post‐Hepatectomy Liver Failure.

	Overall *N* (%)	Grade A *n* (%)	Grade B/C *N* (%)	None *N* (%)	*p* value
**Sex**					< 0.001
Female	5454 (48.8)	159 (2.9)	259 (4.7)	5036 (92.3)	
Male	5,713 (51.2)	273 (4.8)	433 (7.6)	5007 (87.6)	
**Age**					< 0.001
18–44	1670 (15.0)	65 (3.9)	46 (2.8)	1559 (93.4)	
45–64	4848 (43.4)	176 (3.6)	268 (5.5)	4404 (90.8)	
65–74	3202 (28.7)	125 (3.9)	239 (7.5)	2838 (88.6)	
75 and older	1447 (13.0)	66 (4.6)	139 (9.6)	1242 (85.8)	
**Race**					< 0.001
White	6603 (59.1)	218 (3.3)	370 (5.6)	6015 (91.1)	
Asian/Other	3697 (33.1)	188 (5.1)	282 (7.6)	3227 (87.3)	
Black	867 (7.8)	26 (3.0)	40 (4.6)	801 (92.4)	
**ASA Class**					< 0.001
1–2	2685 (24.0)	96 (3.6)	110 (4.1)	2479 (92.3)	
3	7512 (67.3)	289 (3.8)	475 (6.3)	6748 (89.8)	
4	970 (8.7)	47 (4.8)	107 (11.0)	816 (84.1)	
**Bleeding Disorder**					< 0.001
No	10,843 (97.1)	418 (3.9)	653 (6.0)	9772 (90.1)	
Yes	324 (2.9)	14 (4.3)	39 (12.0)	271 (83.6)	
**Ascites**					< 0.001
No	11,112 (99.5)	431 (3.9)	679 (6.1)	10,002 (90.0)	
Yes	55 (0.5)	1 (1.8)	13 (23.6)	41 (74.5)	
**Bilirubin** > **1.2 mg/dL**					< 0.001
No	10,238 (91.7)	391 (3.8)	562 (5.5)	9285 (90.7)	
Yes	929 (8.3)	41 (4.4)	130 (14.0)	758 (81.6)	
**AST** > **40 U/L**					< 0.001
No	8404 (75.3)	288 (3.4)	406 (4.8)	7710 (91.7)	
Yes	2763 (24.7)	144 (5.2)	286 (10.4)	2333 (84.4)	
**Albumin** < **3.4 g/dL**					< 0.001
No	9856 (88.3)	386 (3.9)	532 (5.4)	8938 (90.7)	
Yes	1311 (11.7)	46 (3.5)	160 (12.2)	1105 (84.3)	
**Portal Vein Embolization**					< 0.001
No	10,206 (91.4)	374 (3.7)	577 (5.7)	9,255 (90.7)	
Yes	961 (8.6)	58 (6.0)	115 (12.0)	788 (82.0)	
**Biliary Stent Placement**					< 0.001
Unknown	99 (88.7)	1 (1.0)	7 (7.1)	91 (91.9)	
No/Unknown	10,025 (89.8)	431 (3.9)	268 (5.5)	5007 (87.6)	
Yes	1142 (10.2)	62 (5.4)	178 (15.6)	902 (79.0)	
**Liver Texture**					< 0.001
Normal	3344 (29.9)	126 (3.8)	172 (5.1)	3046 (91.1)	
Abnormal	2841 (25.4)	130 (4.6)	248 (8.7)	2463 (86.7)	
Unknown	4982 (44.6)	176 (3.5)	272 (5.5)	4534 (91.0)	
**Resection**					< 0.001
Right Hepatectomy	5216 (46.7)	278 (5.3)	374 (7.2)	4564 (87.5)	
Left Hepatectomy	3106 (27.8)	39 (1.3)	49 (1.6)	3018 (97.2)	
Trisegmentectomy	2845 (25.5)	115 (4.0)	269 (9.5)	2461 (86.5)	
**Pathology**					< 0.001
Benign	1765 (15.8)	35 (2.0)	49 (2.8)	1681 (95.2)	
Secondary Metastasis	4724 (42.3)	178 (3.8)	242 (5.1)	4304 (91.1)	
Cholangiocarcinoma	2018 (18.1)	85 (4.2)	217 (10.8)	1716 (85.0)	
HCC	1910 (17.1)	95 (5.0)	131 (6.9)	1684 (88.2)	
Unknown	417 (3.7)	22 (5.3)	12 (2.9)	383 (91.8)	
Other Primary	333 (3.0)	17 (5.1)	41 (12.3)	275 (82.6)	
**Tumor Size**					0.004
> 5 cm	1725 (15.4)	68 (3.9)	104 (6.0)	1553 (90.0)	
2–5 cm	1971 (17.7)	77 (3.9)	92 (4.7)	1802 (91.4)	
< 2 cm	831 (7.4)	30 (3.6)	36 (4.3)	765 (92.1)	
Unknown	6640 (59.4)	257 (3.9)	460 (6.9)	5923 (89.2)	
**Biliary Reconstruction**					< 0.001
No/Unknown	9612 (86.1)	344 (3.6)	444 (4.6)	8824 (91.8)	
Hepaticojejunostomy	1555 (13.9)	88 (5.7)	248 (15.9)	1219 (78.4)	
**Colorectal Resection**					0.148
No	10,949 (98.0)	422 (3.9)	672 (6.1)	9855 (90.0)	
Yes	218 (2.0)	10 (4.6)	20 (9.2)	188 (86.2)	

Patients treated at high volume centers were similar in age and biologic sex distribution to those at lower volume institutions, though high volume centers treated fewer white patients (53.9% vs. 70.5%), slightly fewer black patients (6.7% vs. 9.5%), and more patients identifying as Asian or other racial heritage (39.1% vs. 20.0%) (Table 2). High volume institutions had a median annual MH volume of 21.9 (range 13.6–79.3) and accounted for 68.5% (*n* = 7651) of all MHs (Table 3). High volume centers performed right hepatectomies (47.6% vs. 44.8%) and hepaticojejunostomies (14.9% vs. 11.9%) slightly more frequently than low volume centers. Similarly, portal vein embolization (9.7% vs. 6.3%) and preoperative stent placement (10.9% vs. 8.8%) were more frequent at high volume centers.

**Table 2:** Patient Characteristics by Hospital Major Hepatectomy Volume Tier.

	Low Volume	High Volume	*p* value
**Sex**			0.9748
Female	1718 (48.9)	3736 (48.8)	
Male	1798 (51.1)	3915 (51.2)	
**Age**			0.0571
18–44	500 (14.2)	1170 (15.3)	
45–64	1517 (43.1)	3331 (43.5)	
65–74	1063 (30.2)	2139 (28.0)	
75 up	436 (12.4)	1011 (13.2)	
**Race**			< 0.0001
White	2478 (70.5)	4125 (53.9)	
Asian/Other	703 (20.0)	2994 (39.1)	
Black	335 (9.5)	532 (7.0)	
**ASA Class**			< 0.0001
1–2	871 (24.8)	1814 (23.7)	
3	2402 (68.3)	5110 (66.8)	
4	243 (6.9)	727 (9.5)	
**Bleeding Disorder**			0.467
No	3408 (96.9)	7435 (97.2)	
Yes	108 (3.1)	216 (2.8)	
**Ascites**			0.842
No	3498 (99.5)	7614 (99.5)	
Yes	18 (0.5)	37 (0.5)	
**Bilirubin** > **1.2**			0.113
No	3245 (92.3)	6993 (91.4)	
Yes	271 (7.7)	658 (8.6)	
**AST** > **40**			0.815
No	2651 (75.4)	5753 (75.2)	
Yes	865 (24.6)	1898 (24.8)	
**Albumin** < **3.4**			0.811
No	3107 (88.4)	6749 (88.2)	
Yes	409 (11.6)	902 (11.8)	
**Portal Vein Embolization**			< 0.0001
No	3295 (93.7)	6911 (90.3)	
Yes	221 (6.3)	740 (9.7)	
**Stent Placement**			0.001
No/Unknown	3208 (91.2)	6817 (89.1)	
Yes	308 (8.8)	834 (10.9)	
**Liver Texture**			< 0.0001
Normal	946 (26.9)	2398 (31.3)	
Abnormal	890 (25.3)	1951 (25.5)	
Unknown	1680 (47.8)	3302 (43.2)	
**Resection**			0.0004
Left Hepatectomy	1064 (30.3)	2042 (26.7)	3106
Right Hepatectomy	1575 (44.8)	3641 (47.6)	5216
Trisegmentectomy	877 (24.9)	1968 (25.7)	2845
**Pathology**			0.0002
Benign	594 (16.9)	1171 (15.3)	
Secondary Metastasis	1476 (42.0)	3248 (42.5)	
Cholangiocarcinoma	571 (16.2)	1447 (18.9)	
HCC	652 (18.5)	1258 (16.4)	
Unknown	113 (3.2)	304 (4.0)	
Other Primary	110 (3.1)	223 (2.9)	
**Tumor Size**			0.2308
> 5 cm	535 (15.2)	1190 (15.6)	
2‐5 cm	609 (17.3)	1362 (17.8)	
< 2 cm	240 (6.8)	591 (7.7)	
Unknown	2132 (60.6)	4508 (58.9)	
**Biliary Reconstruction**			< 0.0001
No/Unknown	3099 (88.1)	6513 (85.1)	
Hepaticojejunostomy	417 (11.9)	1138 (14.9)	
**Colorectal Resection**			0.958
No	3447 (98.0)	7502 (98.1)	
Yes	69 (2.0)	149 (1.9)	

**Table 3:** Rates of Post Hepatectomy Liver Failure (PHLF) based on Hospital Major Hepatectomy (MH) Volume.

Volume Quartil^a^	Total Patients	Median Annual MH Volume (Range)	No PHLF (%)	Any PHLF (%)	Grade A (%)	Grade B/C
4th	7,651	21.9 (13.6–79.3)	6,749 (88.2)	902 (11.8)	368 (4.8)	534 (7.0)
3rd	2,421	8.8 (5.8–13.5)	2,255 (93.1)	166 (6.9)	50 (2.1)	116 (4.8)
2nd	877	4.0 (2.5–5.8)	829 (94.5)	48 (5.5)	13 (1.5)	35 (4.0)
1st	218	1.6 (1.0–2.4)	210 (96.3)	8 (3.7)	1 (0.5)	7 (3.2)
Total	11,167		10,043 (89.9)	1,124 (10.1)	432 (3.9)	692 (6.2)

a Each volume quartile contains 44 hospitals (*N* = 176).

The overall incidence of PHLF after MH was 10.1%. Lower volume hospitals had lower incidence of PHLF after MH. PHLF was observed in 3.7% of patients in the lowest quartile and 11.8% in the highest quartile. Across all volume tiers, Grade B or C PHLF (6.2% overall) occurred more frequently than Grade A (3.9% overall) (Table 3, Table 4). Multivariable logistic regression revealed a significantly increased odds of PHLF at high volume hospitals compared to low volume hospitals (adjusted odds ratio [aOR]=1.73, 95% Confidence Interval (CI) 1.17–2.57, *p* = 0.007) (Supplement B).

**Table 4:** Rates of six 30‐day outcomes based on major hepatectomy (MH) volume status.

All	Total	Grade A	Grade B/C	None
Mortality	304 (2.7)	8 (2.6)	162 (53.3)	134 (44.1)
Morbidity	2617 (23.4)	162 (6.2)	520 (19.9)	1935 (73.9)
Length of Stay > 75th Percentile	3424 (30.7)	248 (7.2)	501 (14.6)	2675 (78.1)
Return to OR	490 (4.4)	37 (7.6)	154 (31.4)	299 (61.0)
Postoperative Invasive Intervention	1594 (14.3)	115 (7.2)	357 (22.4)	1122 (70.4)
Readmission	1404 (12.6)	97 (6.9)	153 (10.9)	1154 (82.2)
**High Volume** a				
Mortality	215 (2.8)	7 (3.3)	116 (54.0)	92 (42.8)
Morbidity	1825 (23.9)	134 (7.3)	395 (21.6)	1296 (71.0)
Length of Stay > 75th Percentile	2374 (31.0)	199 (8.4)	386 (16.3)	1789 (75.4)
Return to OR	351 (4.6)	29 (8.3)	123 (35.0)	199 (56.7)
Postoperative Invasive Intervention	1118 (14.6)	96 (8.6)	278 (24.9)	744 (66.5)
Readmission	957 (12.5)	80 (8.4)	120 (12.5)	757 (79.1)
**Low Volume^a^**				
Mortality	89 (2.5)	1 (1.1)	46 (51.7)	42 (47.2)
Morbidity	792 (22.5)	28 (3.5)	125 (15.8)	639 (80.7)
Length of Stay > 75th Percentile	1050 (29.9)	49 (4.7)	115 (11.0)	886 (84.4)
Return to OR	139 (4.0)	8 (5.8)	31 (22.3)	100 (71.9)
Postoperative Invasive Intervention	476 (13.5)	19 (4.0)	79 (16.6)	378 (79.4)
Readmission	447 (12.7)	17 (3.8)	33 (7.4)	397 (88.8)

a High‐volume hospitals were ≥ 75th percentile (quartile 4) for average annual MH volume and Low‐volume hospitals were < 75th percentile (quartiles 1‐3)

PHLF was significantly associated with other adverse postoperative outcomes in both high volume and low volume institutions. The odds of 30‐day mortality were dramatically increased by the presence of grade B or C PHLF in all hospitals. This association was more dramatic in low‐volume hospitals (aOR 26.0, 95% CI 13.7–49.7) than in high‐volume centers (aOR 13.3, 95% CI 8.9–19.8). Similar trends were observed for 30‐day morbidity in both low‐volume (aOR 11.2, 95% CI 7.0–17.7) and high‐volume hospitals (aOR 8.5, 95% CI 6.1–11.9). Though grade B or C PHLF was associated with elevated odds of LOS > 75th percentile at both low and high‐volume centers, grade A PHLF demonstrated a much greater association with this outcome in low‐volume hospitals (aOR 8.06, 95% CI 4.53–14.3) than high‐volume centers (aOR 2.82, CI 2.10–3.78). Similarly, PHLF of all grades significantly increased the odds of ROR and other invasive postoperative interventions at both low and high‐volume centers. Though generally larger effect sizes were observed at low volume institutions, the substantial overlap of confidence intervals for these odds ratios generated from separate models suggests these differences did not reach statistical significance (Table 5).

**Table 5:** Multivariable logistic regression adjusted odds ratios for six 30‐day outcomes.

		Low‐Volume^c^	High‐Volume
Outcome^b^	PHLF	aOR (95% CI)^a^	aOR (95% CI)
Mortality	A	1.15 (0.18–7.40)	1.06 (0.41–2.72)
	B/C	26.05 (13.65–49.71)	13.26 (8.90–19.75)
Morbidity	A	3.14 (1.89–5.22)	1.99 (1.41–2.81)
	B/C	11.16 (7.04–17.70)	8.47 (6.06–11.85)
Readmission	A	2.52 (1.37–4.61)	2.01 (1.54–2.63)
	B/C	1.33 (0.80–2.21)	1.71 (1.28–2.28)
Length of Stay > 75th Percentile	A	8.06 (4.53–14.34)	2.82 (2.10–3.78)
	B/C	4.46 (2.82–7.04)	4.51 (3.29–6.18)
Return to OR	A	3.81 (2.02–7.20)	2.33 (1.58–3.45)
	B/C	6.19 (3.74–10.22)	7.64 (5.76–10.13)
Postoperative Invasive Intervention	A	3.29 (1.76–6.15)	2.55 (1.84–3.53)
	B/C	5.43 (3.59–8.23)	6.42 (5.04–8.17)

a Reference category for all listed adjusted odds ratios (aOR) was patients without Post Hepatectomy Liver Failure (PHLF)

b Models were constructed separately for the low‐volume and high‐volume groups. Additional covariates within these models included age, sex, race, ASA class, bleeding disorder, ascites, bilirubin > 1.2 mg/dL, AST > 40 U/L, albumin < 3.4 g/dL, portal vein embolization, biliary stent placement, liver texture, extent of resection, pathology, tumor size, biliary reconstruction, and concomitant colorectal resection.

^c^ Low volume hospitals were < 75th percentile (quartiles 1–3) and high‐volume hospitals were ≥ 75th percentile (quartile 4) for average annual MH volume.

## 4 Discussion

The association of high surgical volume and improved outcomes has been widely documented, though these evaluations have typically focused on mortality and LOS. This analysis evaluated the association between hospital MH volume, incident PHLF, and subsequent 30‐day postoperative outcomes. To the best of our knowledge, this is the first study to examine PHLF and subsequent adverse outcomes in relation to hospital‐level MH volume.

High volume hospitals demonstrated higher unadjusted rates of all grades of PHLF, with significantly increased odds of PHLF compared to lower volume centers after adjusting for factors previously associated with this outcome [42, 43]. We do not anticipate these findings to be reflective of inferior care at higher institutions, given the abundant evidence demonstrating improved outcomes for gastrointestinal oncologic surgery in high‐volume settings which support trends to shift hepatobiliary care for complex diseases to specialized referral centers [44, 45, 46, 47]. Rather, the increased frequency of PHLF at high volume centers observed in this study is more likely the result of advanced disease in complex patients cared for at these centers. This may be evidenced by the greater amount of cholangiocarcinoma (18.9 vs. 16.2%) and hepaticojejunostomy (13.9 vs. 11.9%) seen at higher volume centers, though this detail was not an explicit focus of this study. Variables such as predicted future liver remnant may also reflect the complexity of patients at high volume institutions, though this variable is not included within this dataset.

Other findings were consistent with previous literature. The odds of mortality in patients with grade B or C PHLF were significantly increased compared to those without PHLF across all sites, consistent with existing literature supporting the strong association of grade B and C PHLF with increased mortality [29, 43]. Similarly across all hospitals, grade A PHLF increased the odds of morbidity, readmission, prolonged LOS, ROR, and invasive postoperative intervention compared to patients without PHLF ‐ consistent with recent work establishing grade A PHLF as a distinct, clinically significant entity [43].

Despite higher observed rates of PHLF at high‐volume institutions, OR for subsequent adverse outcomes were lower at these institutions compared to lower volume institutions. However, these comparisons were not statistically significant. This may be a result of the uniformly devastating nature of this complication, with limited treatment options beyond supportive therapies. In rare instances where a correctable etiology, such as inflow or outflow thrombosis, is identified [4], treatment modalities, such as anticoagulation or interventional radiology, are unlikely to be disparate based on MH volume, as one could reasonably expect hospitals performing elective MHs to have appropriately resourced interventional radiology and surgical intensive care. Nevertheless, given the severe and often catastrophic consequences of this outcome, procedures associated with elevated risk may be best concentrated at highly resourced institutions where multidisciplinary expertise and perioperative support are readily available.

These findings highlight the clinical importance of this outcome, given its continued prevalence even among highly specialized, well‐resourced centers. Efforts to improve complex surgical cancer care, including registry participation, quality collaboratives, and cancer‐specific accreditation, should prioritize PHLF as an important mediator of adverse outcomes and potential target for quality improvement. The ACS NSQIP Hepatopancreaticobiliary Collaborative is one such procedure‐based collaborative with the opportunity to leverage this data to improve patient care. Our work highlights the significant consequences of PHLF regardless of hospital MH volume, and the importance of monitoring this outcome at the institutional level, given higher frequency among high‐volume and ostensibly better‐resourced institutions.

There are notable limitations to this study. First, in calculating average annual hospital MH volume, years in which no MH were recorded were considered non‐participating years. This may have mildly skewed the calculated average at the hospital level in favor of lower volume hospitals. However, this would have the greatest effect on the lowest volume hospitals, which were aggregated with second and third quartiles for this analysis. We therefore anticipate a limited effect on our findings. Further, assessing hospital volume within the ACS NSQIP database reflects only NSQIP sampling volume, which has the propensity to underestimate surgical volumes. This would disproportionately affect high volume hospitals. Nevertheless, we expect the highest volume hospitals would have remained in the top quartile even if pure census volumes were obtained. To accommodate for this, we compared the uppermost quartile to the bottom three quartiles. Additionally, this dataset reflects the voluntary participation of institutions, resulting in some potential for sampling bias and small limitations to broader generalizability. Also, the ACS NSQIP collects and reports 30‐day postoperative outcomes, limiting our scope of analysis to this timeframe. Finally, greater risk of PHLF at higher‐volume institutions may be a function of variables not accounted for in the analysis, or variability in the included variables across hospital volume as discussed above. Further characterization through, for example, interaction terms was not included in our analysis, though may better be targeted by further study. Nevertheless, we anticipate small limitations on analysis of outcomes based on grade of PHLF.

## 5 Conclusion

Higher annual major hepatectomy volume is associated with increased incidence of PHLF, which remains a devastating complication with increased subsequent adverse events affecting both high and low volume institutions. This outcome remains an important institutional target for quality improvement. Further study is warranted to characterize key factors contributing to this outcome at well‐resourced, high‐volume institutions.

## Conflicts of Interest

The authors declare no conflicts of interest.

## 6 Synopsis

Post‐hepatectomy liver failure is the leading cause of death following major hepatectomy, though its relationship to hospital major hepatectomy volume has not previously been characterized. Higher annual major hepatectomy volume is associated with increased incidence of post‐hepatectomy liver failure, and subsequent adverse events similarly affect both low and high volume institutions.

## Supporting information


**Supporting File:** jso70280‐sup‐0001‐PHLF_SUPPLEMENT_2025_JSO.docx.

## Data Availability

The authors have nothing to report.
