# Supplementary material for: Hospital Volume and Post‐Hepatectomy Liver Failure After Major Hepatectomy
Source: J Surg Oncol. 2026 May 6;134(1):76–85. doi: 10.1002/jso.70280 (PMC13378781; doi:10.1002/jso.70280)
Supplement: Supplementary file 1 — Supporting File: jso70280‐sup‐0001‐PHLF_SUPPLEMENT_2025_JSO.docx. [file JSO-134-76-s001.docx]

**Supplemental Materials**

**Supplement A**

CPT Codes Used to Identify Concurrent Colorectal Resection

44140', '44141', '44143', '44144', '44145', '44146', '44147', '44150','44151', '44160', '44204', '44205', '44206', '44207', '44208', '44210','44155', '44156', '44157', '44158', '44211', '44212', '45110', '45111','45112', '45113', '45114', '45116', '45119', '45120', '45121', '45123','45126', '45130', '45135', '45160', '45395', '45397', '45402', '45550

**Supplement B.** Multivariable logistic regression for Post Hepatectomy Liver Failure.

| **Variable** | **aOR (95% CI)** | **p** |
| --- | --- | --- |
| **Sex** |  |  |
| Female | 0.70 (0.60-0.81) | <.0001 |
| Male | 1.00 (REF) |  |
| **Age** |  |  |
| 18-44 | 1.00 (REF) |  |
| 45-64 | 1.08 (0.90-1.30) | 0.3804 |
| 65-74 | 1.28 (1.03-1.59) | 0.0255 |
| 75 up | 1.64 (1.23-2.20) | 0.0009 |
| **Race** |  |  |
| White | 1.00 (REF) |  |
| Asian/Other | 1.39 (0.93-2.07) | 0.105 |
| Black | 0.97 (0.67-1.37) | 0.852 |
| **ASA Class** |  |  |
| 1-2 | 1.00 (REF) |  |
| 3 | 1.13 (0.88-1.45) | 0.3395 |
| 4 | 1.54 (1.05-2.27) | 0.0272 |
| **Bleeding Disorder** |  |  |
| No | 1.00 (REF) |  |
| Yes | 1.58 (1.20-2.08) | 0.0014 |
| **Ascites** |  |  |
| No | 1.00 (REF) |  |
| Yes | 2.36 (1.34-4.16) | 0.0033 |
| **Bilirubin > 1.2** |  |  |
| No | 1.00 (REF) |  |
| Yes | 1.00 (0.81-1.22) | 0.9676 |
| **AST > 40** |  |  |
| No | 1.00 (REF) |  |
| Yes | 1.43 (1.22-1.69) | <.0001 |
| **Albumin < 3.4** |  |  |
| No | 1.00 (REF) |  |
| Yes | 1.24 (0.97-1.58) | 0.0878 |
| **Portal Vein Embolization** |  |  |
| No | 1.00 (REF) |  |
| Yes | 1.46 (1.16-1.85) | 0.0014 |
| **Stent Placement** |  |  |
| No/Unknown | 1.00 (REF) |  |
| Yes | 1.35 (1.03-1.78) | 0.0317 |
| **Liver Texture** |  |  |
| Normal | 1.00 (REF) |  |
| Abnormal | 1.45 (1.21-1.73) | <.0001 |
| Unknown | 0.98 (0.77-1.25) | 0.8637 |
| **Resection** |  |  |
| Left Hepatectomy | 1.00 (REF) |  |
| Right Hepatectomy | 5.13 (3.85-6.84) | <0.0001 |
| Trisegmentectomy | 4.25 (2.97-6.09) | <0.0001 |
| **Pathology** |  |  |
| Benign | 1.00 (REF) |  |
| Secondary Metastasis | 1.06 (0.58-1.96) | 0.8475 |
| Cholangiocarcinoma | 1.62 (1.20-2.18) | 0.0017 |
| HCC | 1.55 (1.15-2.10) | 0.0046 |
| Unknown | 1.37 (0.84-2.24) | 0.2078 |
| Other Primary | 1.94 (1.38-2.72) | 0.0002 |
| **Tumor Size** |  |  |
| < 2 cm | 1.00 (REF) |  |
| > 5 cm | 1.24 (0.92-1.66) | 0.1603 |
| 2-5 cm | 1.14 (0.86-1.51) | 0.3756 |
| Unknown | 0.85 (0.46-1.57) | 0.6107 |
| **Biliary Reconstruction** |  |  |
| No/Unknown | 1.00 (REF) |  |
| Hepaticojejunostomy | 2.22 (1.77-2.80) | <.0001 |
| **Colorectal Resection** |  |  |
| No | 1.00 (REF) |  |
| Yes | 1.92 (1.20-3.07) | 0.0068 |
| **Hospital Volume** |  |  |
| Low Volume (<75th Percentile) | 1.00 (REF) |  |
| High Volume (≥75th Percentile) | 1.73 (1.17-2.57) | 0.0067 |

aOR = adjusted odds ratio, 95% CI = 95% Confidence In
